# A Combination of Receptor-Binding Domain and N-Terminal Domain Neutralizing Antibodies Limits the Generation of SARS-CoV-2 Spike Neutralization-Escape Mutants

**DOI:** 10.1128/mBio.02473-21

**Published:** 2021-10-05

**Authors:** Denise Haslwanter, M. Eugenia Dieterle, Anna Z. Wec, Cecilia M. O’Brien, Mrunal Sakharkar, Catalina Florez, Karen Tong, C. Garrett Rappazzo, Gorka Lasso, Olivia Vergnolle, Ariel S. Wirchnianski, Robert H. Bortz, Ethan Laudermilch, J. Maximilian Fels, Amanda Mengotto, Ryan J. Malonis, George I. Georgiev, Jose A. Quiroz, Daniel Wrapp, Nianshuang Wang, Kathryn E. Dye, Jason Barnhill, John M. Dye, Jason S. McLellan, Johanna P. Daily, Jonathan R. Lai, Andrew S. Herbert, Laura M. Walker, Kartik Chandran, Rohit K. Jangra

**Affiliations:** a Department of Microbiology and Immunology, Albert Einstein College of Medicine, New York, New York, USA; b Adimab LLC, Lebanon, New Hampshire, USA; c U.S. Army Medical Research Institute of Infectious Diseases, Frederick, Maryland, USA; d Department of Chemistry and Life Science, United States Military Academy at West Point, West Point, New York, USA; e Department of Biochemistry, Albert Einstein College of Medicine, New York, New York, USA; f Division of Infectious Diseases, Department of Medicine, Albert Einstein College of Medicine and Montefiore Medical Center, New York, New York, USA; g Department of Molecular Biosciences, The University of Texas at Austin, Austin, Texas, USA; h Department of Science, Mount St. Mary’s University, Emmitsburg, Maryland, USA; i The Geneva Foundation, Tacoma, Washington, USA; j Department of Radiology and Radiological Services, Uniformed Services University of the Health Sciences, Bethesda, Maryland, USA; k Adagio Therapeutics Inc., Waltham, Massachusetts, USA; Duke University School of Medicine

**Keywords:** COVID-19, NTD, RBD, SARS-CoV-2, antibody, variants of concern

## Abstract

Most known SARS-CoV-2 neutralizing antibodies (nAbs), including those approved by the FDA for emergency use, inhibit viral infection by targeting the receptor-binding domain (RBD) of the spike (S) protein. Variants of concern (VOC) carrying mutations in the RBD or other regions of S reduce the effectiveness of many nAbs and vaccines by evading neutralization. Therefore, therapies that are less susceptible to resistance are urgently needed. Here, we characterized the memory B-cell repertoire of COVID-19 convalescent donors and analyzed their RBD and non-RBD nAbs. We found that many of the non-RBD-targeting nAbs were specific to the N-terminal domain (NTD). Using neutralization assays with authentic SARS-CoV-2 and a recombinant vesicular stomatitis virus carrying SARS-CoV-2 S protein (rVSV-SARS2), we defined a panel of potent RBD and NTD nAbs. Next, we used a combination of neutralization-escape rVSV-SARS2 mutants and a yeast display library of RBD mutants to map their epitopes. The most potent RBD nAb competed with hACE2 binding and targeted an epitope that includes residue F490. The most potent NTD nAb epitope included Y145, K150, and W152. As seen with some of the natural VOC, the neutralization potencies of COVID-19 convalescent-phase sera were reduced by 4- to 16-fold against rVSV-SARS2 bearing Y145D, K150E, or W152R spike mutations. Moreover, we found that combining RBD and NTD nAbs did not enhance their neutralization potential. Notably, the same combination of RBD and NTD nAbs limited the development of neutralization-escape mutants *in vitro*, suggesting such a strategy may have higher efficacy and utility for mitigating the emergence of VOC.

## INTRODUCTION

Severe acute respiratory syndrome coronavirus 2 (SARS-CoV-2) is a member of the family *Coronaviridae* and the causative agent of the ongoing coronavirus disease 2019 (COVID-19) pandemic (1). Over 171 million cases have been officially diagnosed since its first emergence, and >3.6 million people have succumbed to disease (2). Public health measures, along with rapid vaccine development, have helped slow the pandemic in some countries. Moreover, small-molecule inhibitors, antibody-based therapeutics, and convalescent-phase plasma from COVID-19 convalescents have received emergency use authorizations (EUAs) (3). Recently, multiple virus variants of concern (VOC), some carrying neutralizing antibody (nAb)-resistant mutations that are associated with increased transmission and fatality rates, have emerged (4). The availability of multiple therapeutic approaches, especially for people who cannot get vaccinated, is essential. There is thus an urgent need to develop therapeutics, especially ones that limit the emergence of neutralization-resistant variants or are more efficient against them as they can help save lives while vaccines are being deployed.

SARS-CoV-2 entry into host cells is mediated by the transmembrane spike (S) glycoprotein, which forms trimeric spikes protruding from the viral surface (5). Each monomer, 180 to 200 kDa in size, comprises S1 and S2 subunits that are generated by posttranslational cleavage by the host enzyme furin. The S1 subunit is composed of two domains, an N-terminal domain (NTD) and a C-terminal domain (CTD). The CTD functions as the receptor-binding domain (RBD) for the entry receptor, human angiotensin-converting enzyme 2 (hACE2) (6, 7). The role of the NTD for SARS-CoV-2 is unclear, but it has been proposed in other coronaviruses to play roles in recognizing specific sugar moieties during attachment and regulating the prefusion-to-postfusion transition of the S protein (8–10). The S2 subunit is composed of the fusion peptide, heptad repeats 1 and 2, a transmembrane domain, and a cytoplasmic tail. Aided by hACE2-binding and host cathepsin- and/or transmembrane protease serine 2 (TMPRSS2)-mediated proteolytic processing, S2 undergoes extensive conformational rearrangement to insert its fusion peptide into the host membrane and mediate the fusion of host and viral membranes (6, 7).

The S protein is the major target of nAbs, the production of which is a key correlate of protection following virus infection and vaccination (11–14). Due to their potential to interfere with hACE2 interaction and to efficiently neutralize virus infection, RBD-specific antibodies have been the main focus of human monoclonal antibody (MAb)-based therapeutics (13, 15–20). We recently described the memory B-cell repertoire of a convalescent SARS donor and isolated multiple RBD-specific antibodies that neutralize and protect against SARS-CoV, SARS-CoV-2, and WIV1 viruses (19, 20). Since that time, multiple RBD-targeting MAbs have received emergency use authorizations by the U.S. FDA. However, the widespread circulation of nAb-resistant variants has led to the withdrawal of EUAs for some nAb monotherapies (21), highlighting the need to develop combination-nAb therapies that can treat SARS-CoV-2 variants and reduce the probability of mutational escape. In fact, a few of the VOC carry mutations in some of the major neutralizing epitopes in the RBD as well as the NTD (22).

Recently, multiple NTD MAbs with potent neutralizing activity have been described (17, 23–28). As combinations of MAbs targeting distinct epitopes and mechanisms of action have been successfully used against other viruses (29, 30), cocktails of RBD and NTD MAbs have been proposed and recently showed promise against SARS-CoV-2 *in vitro* and *in vivo* (23, 24).

To evaluate the effect of combining nAbs targeting the RBD and the NTD, here, we mined the memory B-cell repertoires of four convalescent COVID-19 donors with high serum neutralization and spike-specific antibody titers. By sorting spike-reactive single B cells, we isolated 163 MAbs targeting S. Further, we evaluated their neutralization capacity against authentic SARS-CoV-2 and a self-replicating vesicular stomatitis virus carrying SARS-CoV-2 S protein (rVSV-SARS2) (31). We downselected the top RBD- and NTD-targeting neutralizers and used multiple approaches to map their epitopes. As described recently (32, 33), we observed that neutralization-escape rVSV-SARS2 mutants of the NTD-targeting MAb were resistant to neutralization by COVID-19 convalescent donor sera, suggesting that natural variants in the NTD could, at least in part, escape the antibody response. Although a combination of the NTD- and RBD-targeting MAbs did not neutralize the virus more efficiently, it limited the emergence of neutralization-escape spike mutants, underscoring the utility of combination therapy.

## RESULTS

### SARS-CoV-2 induces robust and diverse memory B-cell response in convalescent patients.

To characterize the B-cell responses induced by SARS-CoV-2 infection, we sampled peripheral blood mononuclear cells (PBMCs) from four adult patients (EMC 5, 9, 10, and 15) at the Montefiore Medical Center in the Bronx (Einstein-Montefiore COVID-19). They were all previously healthy individuals who developed mild COVID-19. SARS-CoV-2 infection in their nasopharynx was confirmed by a positive reverse transcription-quantitative PCR (RT-qPCR) test in the first week of March 2020. All four patients had convalescent-phase blood drawn to collect serum and PBMCs at least 2 weeks after all symptoms had resolved on 31 March 2020 (see Fig. S1A in the supplemental material). Serum samples of all four donors displayed reciprocal serum neutralization half-maximal inhibitory concentration (IC_50_) titers of >118 against rVSV-SARS2.

10.1128/mBio.02473-21.1 FIG S1 (A) Details of SARS-CoV-2 convalescent donors. Neutralization IC_50_ values are shown as reciprocal serum titers. (B and C) Identification and sorting of SARS-COV-2 S-reactive memory B cells in COVID-19 convalescent patients. (B) Representative gating strategy for the identification of class-switched SARS-CoV-2 S-reactive B cells that were double positive for PE- and APC-labeled SARS-CoV-2 S-2P spike protein tetramers. (C) Percentage of class-switched SARS-CoV-2 S-reactive B cells in naive and convalescent donors. The gated populations were single-cell sorted for cloning of the VH and VL genes. A naive donor sample drawn and processed in August 2019 is shown for comparison. Download FIG S1, TIF file, 2.8 MB. Copyright © 2021 Haslwanter et al. 2021 Haslwanter et al. https://creativecommons.org/licenses/by/4.0/ This content is distributed under the terms of the Creative Commons Attribution 4.0 International license.

**FIG 1:**
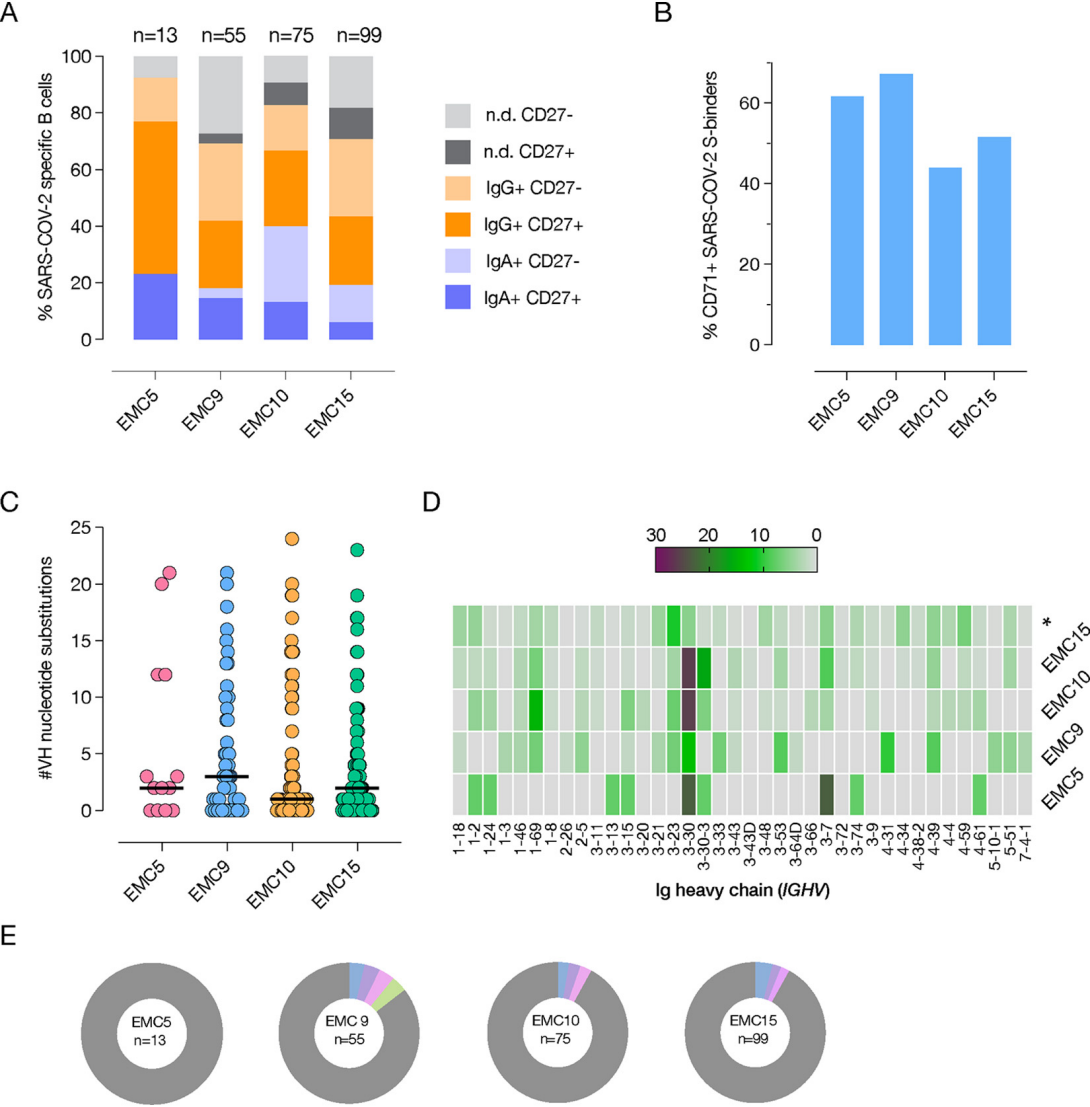
SARS-CoV-2 induces robust and diverse memory B-cell response in COVID-19 convalescent patients. (A and B) Relative proportion of immunoglobulin isotype and classical memory B-cell surface marker CD27 expression (A) or activation marker CD71 expression (B) on B cells from which SARS-CoV-2 S-specific MAbs were derived. n.d., not determined. (C) Number of nucleotide substitutions in the VH genes of the MAbs isolated from the indicated donors. (D) VH germ line gene (*IGHV*) usage in SARS-CoV-2 S-specific MAbs; * indicates the frequency of germ line gene usage in unselected human repertoire (53) for comparison. (E) Clonal lineage analysis of SARS-CoV-2 S-specific MAbs for each of the donors. Donor ID and the total number of clones are indicated in the center of each pie. Unique clones are shown in gray; colored slices indicate expanded clones.

For each donor, we single-cell sorted SARS-CoV-2 S-reactive class-switched (CD19^+^ IgM^−^ IgD^−^) B cells, which ranged in frequency between 0.6 and 1.2% across the donors (Fig. S1B and C). Index-sorting analysis revealed that the S-specific B cells were predominantly IgG^+^, and the majority expressed the classical memory B-cell marker CD27 (41 to 76%) (Fig. 1A). Additionally, 44 to 67% expressed the activation/proliferation marker CD71 (Fig. 1B), consistent with the early time postinfection. Antibodies from all four donors showed similar levels of somatic hypermutation, as evidenced by the median number of nucleotide substitutions in the heavy-chain variable region that are consistent with the early time point postinfection (range 1 to 3) (Fig. 1C). Although variable heavy (VH) germ line genes such as VH3-30 and VH3-30-3 were overrepresented in all individuals as has been seen previously (27), less than 5% of clones were derived from clonally expanded lineages (Fig. 1D and E). Altogether, these results indicate a robust and diverse early memory B-cell response to SARS-CoV-2 infection in each donor and are consistent with previous studies (34–36).

### RBD- and NTD-targeting MAbs are potent neutralizers of SARS-CoV-2.

**FIG 2:**
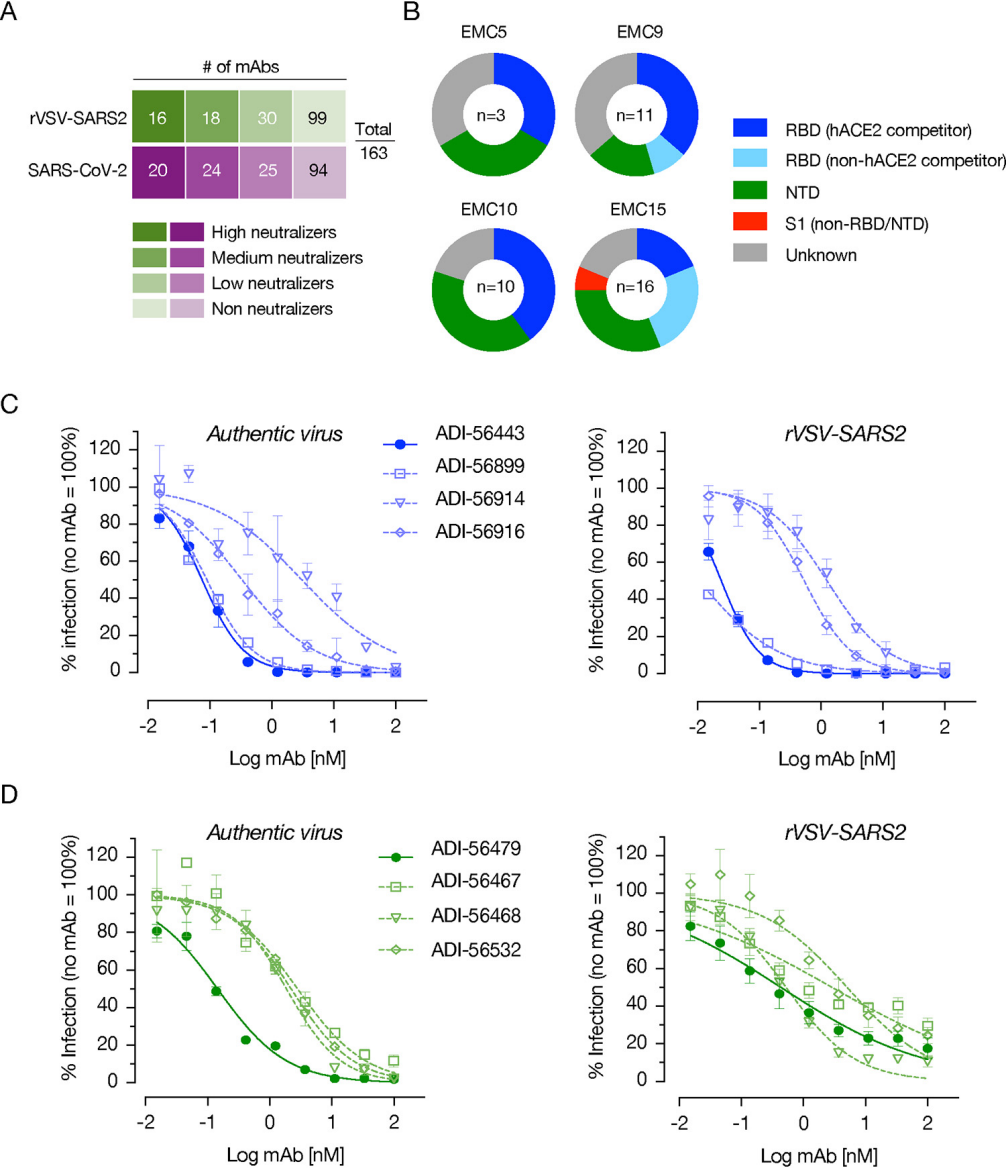
RBD- as well as NTD-targeting MAbs are potent neutralizers of SARS-CoV-2. (A) Screening of high-affinity SARS-CoV-2 spike protein-binding MAbs for their neutralization capacity against rVSV-SARS2 and authentic SARS-CoV-2. The MAbs were divided into high neutralizers (80% neutralization efficacy), medium neutralizers (50 to 80%), low neutralizers (30 to 50%), and nonneutralizers (<30%) based on their capacity to neutralize rVSV-SARS2 at 10 nM or authentic virus at 100 nM antibody concentration. The number of MAbs in each group is indicated in the corresponding boxes. (B) Proportion of the 40 best nAbs, from each donor, targeting each of the indicated domains/regions of the spike protein. (C and D) Neutralization curves of each of the top four RBD- (C) and non-RBD-targeting (D) MAbs. The data were curve-fitted using a nonlinear regression (log [inhibitor] versus normalized response, variable slope) to calculate IC_50_ values (average ± SD, *n* = 4 from two independent experiments for rVSV-SARS2 and *n* = 2 from a representative of multiple experiments for the authentic virus).

From the cloned pairs of VH and VL genes of the four donors, we expressed and purified MAbs in a human IgG1 background using our yeast expression platform. These MAbs were screened for their S protein binding ability, and the top 163 MAb binders were evaluated for their neutralizing activity against authentic SARS-CoV-2 and rVSV-SARS2 at 100 nM and 10 nM antibody concentrations in microneutralization assays, respectively. A total of 44 MAbs neutralized authentic virus with more than 50% efficacy, and 34 MAbs did the same for rVSV-SARS2 (Fig. 2A). Based on these data, we selected the top 40 nAbs for further analyses and used biolayer interferometry (BLI) to identify the specific domains of the S protein targeted by these nAbs (Fig. 2B). Most of the nAbs mapped to the RBD and included hACE2 competitors and noncompetitors (Fig. 2B). However, we also identified multiple non-RBD binding nAbs, including many that target the NTD (Fig. 2B). Seven of our top 40 MAbs did not bind to the RBD, the NTD, or the S1 subunit. These MAbs are likely to bind to the S2 subunit or an epitope(s) that is not present in the RBD, NTD, or S1 subunit. These MAbs belong to the medium neutralizers group (Fig. 2A) with 62 to 80% neutralization efficacy at 100 nM with the authentic virus (Fig. S2). Finally, we ran 9-point neutralization curves on all 40 nAbs with authentic virus and rVSV-SARS2 to downselect to four each of the most potent RBD- and non-RBD-targeting antibodies (data not shown). The neutralizing profiles of the RBD-targeting MAbs against rVSV-SARS2 and the authentic SARS-CoV-2 were very similar, with IC_50_ neutralization values in the picomolar range for ADI-56443 (75 pM) and ADI-56899 (89 pM) (Fig. 2C). As has been observed previously for NTD-targeting MAbs (25), rVSV-SARS2 neutralization curves for the non-RBD MAbs were shallower, and these MAbs left an unneutralized fraction of the virus (Fig. 2D, right panel). However, there was no unneutralized fraction left with the authentic virus (Fig. 2D, left panel). These apparent differences in neutralization curves of rVSV-SARS2 and the authentic virus with the NTD MAbs may be due to the heterogeneity in the glycosylation patterns of the spike protein (37, 38) and/or differences in spike protein density. Greater susceptibility of rVSV-SARS2 to neutralization by RBD-targeting antibodies (19, 31, 39) could be due to the relatively lower density of spike trimers on the rVSV-SARS2. NTD MAbs may neutralize the virus by engaging epitopes on adjacent trimers whose lower density will make rVSV-SARS2 neutralization less efficient. Nevertheless, ADI-56479 was the best NTD-targeting nAb, with picomolar neutralization IC_50_ values against the authentic virus (144 pM).

10.1128/mBio.02473-21.2 FIG S2 Summary of neutralization capacities and antigenic sites of top 40 MAbs. MAb binding to the SARS-CoV-2 spike protein subunit/domain as determined by BLI is indicated as yes (+), no (-), or not determined (n.d.). Download FIG S2, TIF file, 2.6 MB. Copyright © 2021 Haslwanter et al. 2021 Haslwanter et al. https://creativecommons.org/licenses/by/4.0/ This content is distributed under the terms of the Creative Commons Attribution 4.0 International license.

### Epitope mapping of the most potent RBD and NTD MAbs.

**FIG 3:**
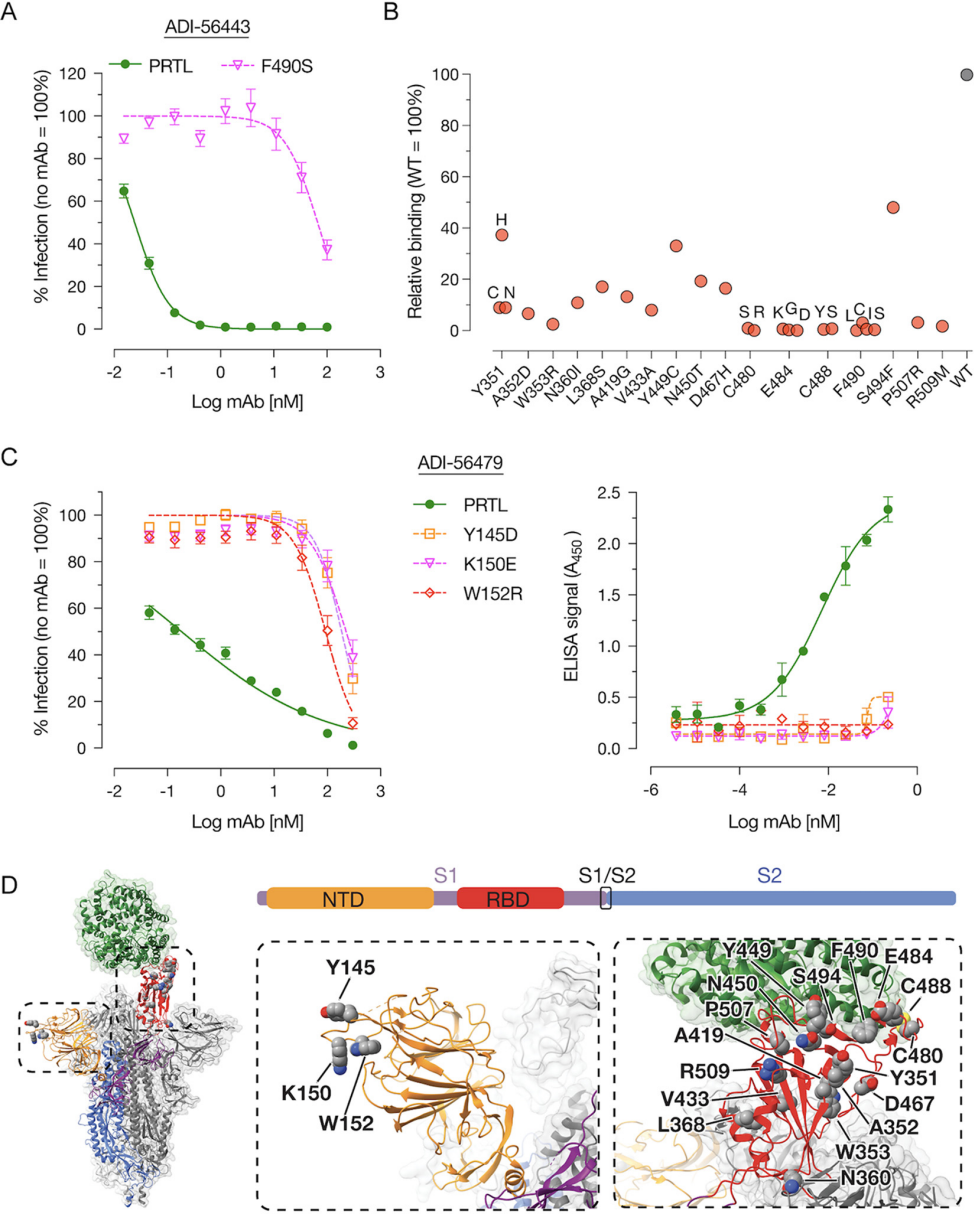
Epitope mapping of RBD- (ADI-56443) and NTD-targeting (ADI-56479) nAbs. (A) Pretitrated amounts of the parental (PRTL) or indicated rVSV-SARS2 mutants were incubated with serial 3-fold dilutions of ADI-56443 at room temperature for 1 h prior to infecting monolayers of Vero cells. After 7 h, cells were fixed, the nuclei were stained, and infected cells were scored by eGFP expression (averages ± SEM, *n* = 4 from 2 independent experiments). (B) Binding capacity of ADI-56443 to a mutagenized library of SARS-CoV-2 RBD point mutants displayed on the surface of yeast cells was measured by flow cytometry. Key residues that led to a loss of ADI-56443 binding are shown (binding to cells displaying WT RBD was set at 100%). Antibody binding was assessed at their EC_80_s for the WT RBD construct. (C) (Left panel) Neutralization capacity of ADI-56479 against pretitrated amounts of parental (PRTL) and indicated rVSV-SARS2 mutants was determined as described above (averages ± SEM, *n* = 4 from 2 independent experiments). (Right panel) ELISA plates coated with the parental or indicated rVSV-SARS2 mutants and binding of biotinylated ADI-56479 to the spike protein was detected by using HRP-conjugated streptavidin. A representative data set from 2 independent experiments is shown here (average ± SD, *n* = 2). (D) (Left panel) An overview of the SARS-CoV-2 S trimer bound to hACE2 (green) (26). For clarity, only the domains of one spike monomer have been colored (S1, purple; NTD, yellow; RBD, red; S2, blue). (Middle and right panels) A closeup view of the NTD (in yellow) and RBD (in red) with amino acid residues important for binding to ADI-56479 (middle panel) and ADI-56443 (right panel) is shown.

To better understand the mechanism of neutralization by our most potent nAbs, we mapped their epitopes by selecting rVSV-SARS2 neutralization-escape mutants. After serially passaging rVSV-SARS2 in the presence of increasing concentrations of the nAbs, we plaque-purified and sequenced resistant viruses to identify the S mutations that confer resistance. For the best RBD MAb (ADI-56443), a change at amino acid position 490 in the S protein (F490S) made rVSV-SARS2 highly resistant (>2,700-fold increase in neutralization IC_50_ value) to this MAb (Fig. 3A). To comprehensively map its epitope, we analyzed the binding capacity of ADI-56443 to a library of SARS-CoV-2 RBD single-amino-acid mutants displayed on the surface of yeast cells by flow cytometry. In addition to F490S, binding of ADI-56443 was completely abolished by C480S/R, E484K/G/D, C488Y/S, and F490L/I/C RBD mutations (Fig. 3B). Mutations at other residues, including S494F in the RBD, also significantly reduced this MAb’s binding (Fig. 3B). Remarkably, residues E484, F490, and S494 are shared with the epitope of MAb LY-CoV555 (bamlanivimab), which received an EUA for COVID-19 treatment (40). The E484K mutation is also present in multiple variants including P.1, P.2, B.1.525, and B.1.351, and viruses carrying this mutation are resistant to the currently used MAb therapy (41, 42). For the NTD MAb (ADI-56479), rVSV-SARS2 neutralization-escape mutations mapped to residues 145 (Y145D), 150 (K150E), and 152 (W152R) in the NTD, and each one of these mutations individually afforded an ∼1,000-fold increase in the neutralization IC_50_ values (Fig. 3C, left panel). This was accompanied by a loss of binding of the ADI-56479 MAb to the mutant spike proteins as determined by enzyme-linked immunosorbent assay (ELISA) using rVSV-SARS2 particles (Fig. 3C, right panel). Consistent with the RBD MAb (ADI-56443) being a competitor of hACE2-spike binding, its epitope partly overlaps the receptor-binding interface (Fig. 3D, right panel). In contrast, all three of the residues in ADI-56479’s epitope are located in the N3 loop of the NTD (Y145, K150, and W152) away from the RBD. Taken together, we have identified two potent neutralizing antibodies that target two distinct domains of the SARS-CoV-2 spike protein.

### rVSV-SARS2 NTD mutants are resistant to neutralization by COVID-19 convalescent-phase sera.

**FIG 4:**
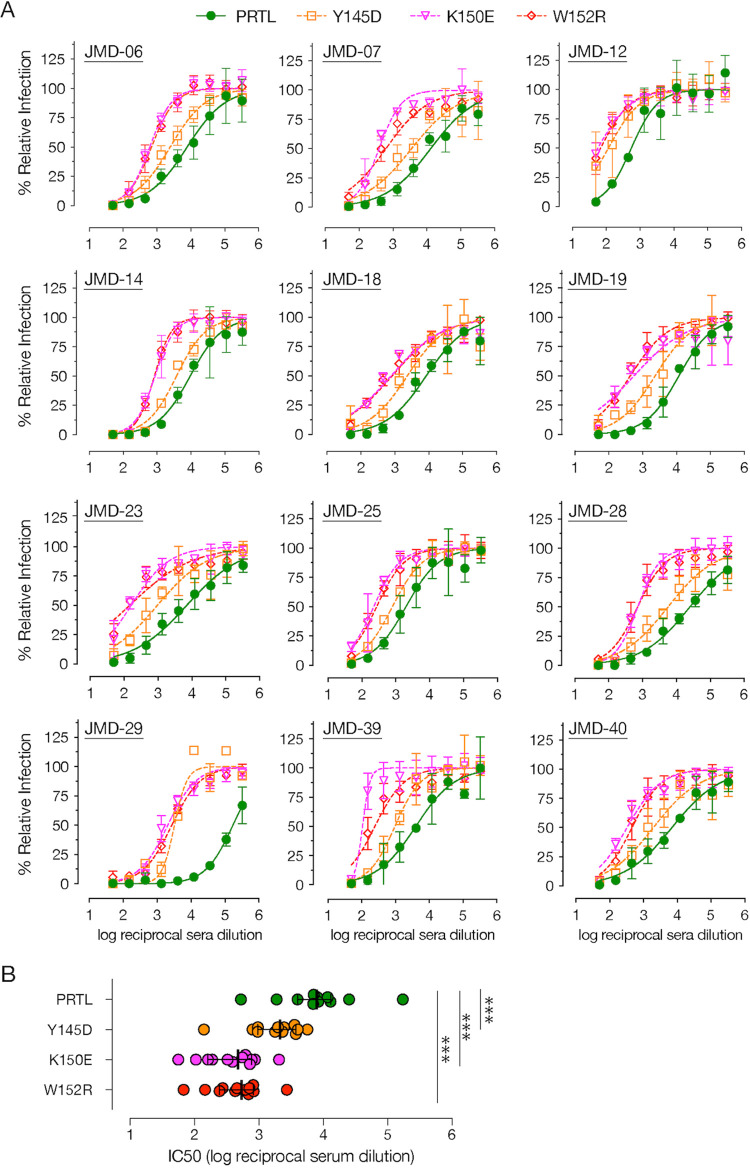
NTD point mutations significantly reduce neutralization of rVSV-SARS2 by convalescent-phase sera. (A) Neutralization of the parental (PRTL) and indicated rVSV-SARS2 mutants with convalescent-phase sera from 12 donors with high nAb titers (reciprocal serum IC_50_ titers of >500). Pretitrated amounts of indicated rVSV-SARS2 were incubated with 3-fold serial dilutions of COVID-19 convalescent-phase sera at room temperature for 1 h and applied to monolayers of Vero cells. After 7 h, cells were fixed and the nuclei were stained. Infected cells were scored by eGFP expression (averages ± SEM, *n* = 4 from 2 independent experiments). (B) Reciprocal serum neutralization IC_50_ titers of all the convalescent-phase sera against parental (PRTL) and mutant rVSV-SARS2 shown in panel A are depicted. The Wilcoxon test was performed to evaluate statistical significance between the neutralization efficacies against the parental and mutant viruses (***, *P* ≤ 0.001).

Although the most potent SARS-CoV-2 nAbs target the RBD (12, 13, 15–20), recent antibody profiling efforts and the emergence of multiple VOC with mutations in the NTD that affect the efficacy of nAbs suggest that NTD-directed antibodies are important for effective control of virus infection (22–24, 43). Given that our NTD MAb’s epitope overlapped the “antigenic supersite” identified by multiple studies recently (23, 24, 27), we hypothesized that this is an immunodominant epitope. Accordingly, we tested if COVID-19 convalescent-phase serum-mediated neutralization of rVSV-SARS2 by human polyclonal sera is altered by the NTD mutations generated in response to the ADI-56479 MAb-driven selection (Fig. 4). Using a set of convalescent-phase sera with high neutralizing activity (reciprocal serum neutralization IC_50_ titers of >500), we observed a significant effect of NTD point mutations on rVSV-SARS2 neutralization (Fig. 4A). Specifically, the sera showed a drop of 3.5-fold (Y145D) to 16-fold (K150E and W152R) in their neutralization IC_50_ titers compared to the parental virus (Fig. 4B). Thus, our data further support a significant role of the NTD-targeting antibodies in the neutralizing activity of SARS-CoV-2-convalescent-phase sera (32, 33).

### Combination of RBD and NTD monoclonal antibodies limits the generation of neutralization-escape mutants.

**FIG 5:**
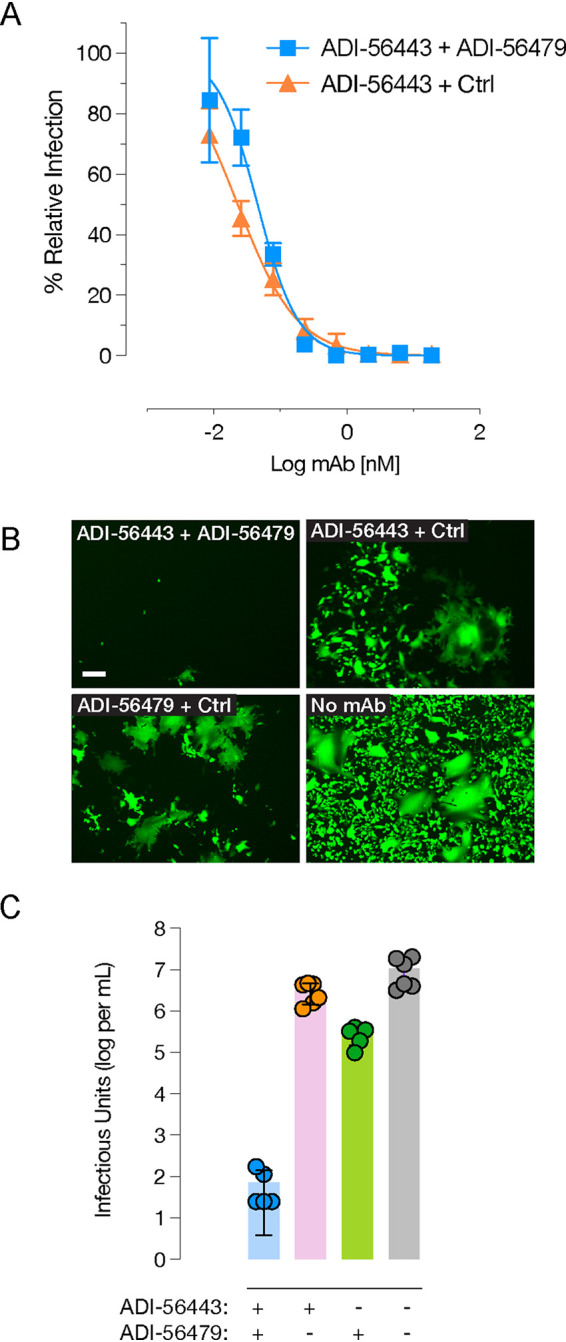
Combining RBD- and NTD-targeting MAbs enhances resistance to neutralization escape. (A) Neutralization of rVSV-SARS2 by RBD-targeting (ADI-56443) MAb in combination with the NTD-targeting (ADI-56479) MAb or control MAb (ADI-15878) (average ± SD, *n* = 5 to 6 from two independent experiments). (B) Representative images of eGFP expression of Vero cells at 24 hpi with rVSV-SARS2 in the presence of IC_90_s of the RBD- (ADI-56443) and NTD-targeting (ADI-56479) MAbs alone or in combination. The no-MAb control is shown for comparison. Bar = 100 μm. (C) Quantitation of rVSV-SARS2 (infectious units per ml) produced from Vero cells in panel B at 48 hpi (average ± SD, *n* = 5 to 6 from two independent experiments).

Since the top RBD (ADI-46443) and NTD (ADI-56479) nAbs bind to two distinct domains of the S protein (Fig. 3D), we reasoned that combining them may enhance their neutralization efficacy. Given the vastly different IC_50_ values of the two MAbs with rVSV-SARS2, we were not able to calculate a classical synergy combination index (CI) analysis based on their equimolar combination at IC_50_ (44). However, we did not observe a significant shift in the rVSV-SARS2 neutralization curve by combining RBD MAb with a fixed amount (3 nM, nearly 10 times its IC_90_ concentration) of the NTD MAb (Fig. 5A). Next, we evaluated if combining the two MAbs, in a 1:1 ratio at their neutralization IC_90_ concentrations, also has an effect on limiting the emergence of rVSV-SARS2 neutralization-escape mutants compared to the single MAbs (Fig. 5B). As expected, rVSV-SARS2 rather easily escaped individual MAbs, reaching a peak titer of 3 × 10^6^ (RBD nAb) and 2.7 × 10^5^ (NTD nAb) infectious units per ml compared to the no-nAb controls (1.2 × 10^7^ infectious units per ml) at 48 h postinfection (Fig. 5C). However, rVSV-SARS2 failed to grow in the presence of the MAb combination (peak titer of 72 infectious units per ml). Thus, in addition to enhancing neutralization efficacy, this MAb combination limits the development of MAb-resistant spike mutants.

## DISCUSSION

All of the SARS-CoV-2 nAbs in phase III clinical trials or clinical use under EUA to treat COVID-19 are RBD specific (12, 13, 15–18, 20). However, potent non-RBD nAbs are present in COVID-19-convalescent patients, and the emergence of NTD mutations that afford resistance to neutralization in multiple SARS-CoV-2 VOC highlights the importance of the NTD as an important target for nAbs. Here, we isolated potent RBD and NTD nAbs from multiple COVID-19 donors and mapped their epitopes in the S protein. We show that neutralization-escape mutations to the NTD nAb significantly reduce the efficacy of polyclonal COVID-19 convalescent-phase sera. Importantly, a combination of RBD and NTD MAbs could effectively limit the emergence of nAb-resistant S mutations.

Consistent with the increasingly recognized importance and prevalence of non-RBD nAbs (43), 12 of our top 40 nAbs targeted the NTD (Fig. 1 and 2). Six of these NTD nAbs were derived from two variable heavy (VH) genes (VH1-24 or VH3-30), suggesting their preponderance in NTD nAbs. Some NTD nAbs require significant somatic hypermutations for their efficacy (23, 27). However, 11 out of the 12 nAbs that we discovered carried ≤3 somatic hypermutations, indicating that they arose at an early time point postinfection. Thus, the nAb response to the NTD is polyclonal, and many potent NTD nAbs require relatively low levels of affinity maturation, as described recently (23).

Although blocking of hACE2-Spike interactions is one of the major mechanisms of virus neutralization, NTD-targeting antibodies with other blocking mechanisms have been reported (23–28). However, their precise mechanisms of action remain unclear. Some appear to inhibit a postattachment phase of virus infection and may block subsequent viral steps in entry and/or promote Fc-mediated effector functions (23, 24). For the distantly related Middle East respiratory syndrome coronavirus (MERS-CoV), the NTD can bear a critical epitope(s) for neutralization (45). Here, we show that a potent NTD nAb (ADI-56479) interacts with residues Y145, K150, and W152 in the NTD (Fig. 3), which are located in the N3 loop of an “antigenic supersite.” Other potent NTD nAbs also recognize the same antigenic supersite (17, 23–25, 27). Recently, Suryadevara et al. (24) showed that mutations F140S and G142D or R158S in the NTD confer resistance to two other NTD nAbs. Notably, our rVSV-SARS2 neutralization-escape variants (Y145D, K150E, and W152R) were significantly resistant to neutralization by convalescent-phase COVID-19 sera (Fig. 4). In addition, mutations at N148S, K150R/E, and S151P in the NTD epitope exhibited reductions in sensitivity to three COVID-19 convalescent-phase plasma samples (32). A deletion at F140 that occurred in response to immune pressure from highly neutralizing convalescent-phase plasma also partially reduced neutralization (33). Interestingly, a VOC, B.1.429, which displays moderate reduction in neutralization against convalescent-phase and postvaccination sera carries a mutation at W152 (46). One of the Indian variants, B.1.617.2, has as well shown deletions at positions 156 and 157, emphasizing that the NTD is acquiring adaptive mutations that counteract the immune response (22, 46). Collectively, these findings underscore the importance of the NTD in virus entry and the potential of NTD mutations to impact the effectiveness of vaccines and nAb therapies.

Combination therapies with RBD and NTD nAbs have been proposed as a strategy to mitigate the emergence of antibody-resistant variants (6, 17, 24, 25, 32). We observed that a combination of RBD and NTD MAbs did not enhance *in vitro* neutralization efficacy relative to the individual MAbs. In particular, the virus was less likely to escape when passaged in the presence of an RBD and NTD nAb cocktail than in the presence of each nAb alone (Fig. 5). This finding is consistent with what has recently been reported with other NTD and RBD nAb combinations (23, 24). Our findings, together with recent publications, provide evidence that some NTD-targeting MAbs can efficiently inhibit SARS-CoV-2 infection. However, given that our top NTD MAb’s epitope overlaps mutations in VOC spikes, neutralization efficacy of this MAb will likely be lower as has been observed for the other NTD MAbs (38, 47–49). While SARS-CoV-2 continues to evolve into multiple VOC, a combination antibody therapy with properly selected combinations of two potent nAbs targeting distinct conserved epitopes could provide an important solution for treating COVID-19 and impede the generation of novel variants, especially in populations that cannot be vaccinated.

## MATERIALS AND METHODS

### Cells and viruses.

The African vervet monkey kidney Vero (CCL-81) cells were cultured in Dulbecco’s modified Eagle’s medium (DMEM high glucose; Gibco) supplemented with 2% heat-inactivated fetal bovine serum (FBS; Atlanta Biologicals), 1% penicillin-streptomycin (Gibco), and 1% Gluta-Max (Gibco). The cells were passaged every 3 to 4 days using 0.05% Trypsin-EDTA solution (Gibco).

All the experiments described here with the authentic SARS-CoV-2 (Washington state isolate MT020880.1) were carried out in biosafety level 3 (BSL-3) laboratories at USAMRIID, Frederick, MD, as per Federal regulations under institutional biosafety committee-approved protocols. Virus stocks were prepared as described previously (31). Sequencing of the virus stock revealed a single mutation (a histidine-to-tyrosine change at amino acid position 655, H655Y) in the spike glycoprotein relative to the reference Washington state isolate.

A plaque-purified rVSV-SARS2 corresponding to the passage 9 (plaque 2 virus) described previously (31) was used for these studies and carries W64R, G261R, A372T, H655Y, and R685 mutations in addition to the c-tail truncation (C1253*) mutation. It is referred to here as the parental (PRTL) virus. Virus stocks were generated by growing the virus on Vero cells. Appropriate approvals from the Environmental Health and Safety Department and the Institutional Biosafety Committee at Albert Einstein College of Medicine were sought for using rVSV-SARS2 at biosafety level 2.

### Collection of COVID-19 convalescent donor blood samples.

Convalescent-phase blood samples were collected from healthy adult patient volunteers who had mild COVID-19 and a positive RT-qPCR test for SARS-CoV-2 in March 2020 in Westchester County, New York. These patients neither were hospitalized nor required oxygen supplementation during illness. All donors recovered and were asymptomatic for at least 14 days prior to venipuncture to collect serum and PBMCs. Serum was centrifuged, aliquoted, and stored at −80°C. Sera were heat inactivated at 56°C for 30 min and stored at 4°C prior to antibody testing. The study protocol was approved by the Institutional Review Board (IRB) of the Albert Einstein College of Medicine (IRB number 2016-6137).

### RT-qPCR to detect SARS-CoV-2 infection.

SARS-CoV-2 RT-qPCR was performed per the CDC protocols (50). Briefly, RNA was isolated from blood and PBMCs using the Quick-RNA viral kit (Zymo). Total RNA was mixed with the respective primers and probes (all purchased from IDT) specific for 2019-nCoV (N1 and N2 assays), SARS-like coronaviruses (N3 assay), and human RNase P (RP assay) together with TaqPath 1-Step RT-qPCR Master Mix CG (ThermoFisher). A plasmid containing the complete nucleocapsid gene from 2019-nCoV (IDT) was used as a positive control. In addition, RNA transcribed from a plasmid containing a portion of the RPP30 gene (IDT) was used as quality control for the RNA isolation. All samples were run and analyzed using the iQ5 device (Bio-Rad).

### rVSV-SARS2 neutralization assay.

Parental and mutant rVSV-SARS2 were generated and used for the microneutralization assay as described previously (31). In short, serum samples or monoclonal antibodies were serially diluted and incubated with virus for 1 h at room temperature. For initial screening, a single concentration of 10 nM MAb was used instead. Serum or antibody-virus mixtures were then added in duplicates or triplicates to 96-well plates (Corning) containing monolayers of Vero cells. After 7 h at 37°C and 5% CO_2_, cells were fixed with 4% paraformaldehyde (Sigma), washed with phosphate-buffered saline (PBS), and stored in PBS containing Hoechst 33342 (Invitrogen). Viral infectivity was measured by automated enumeration of green fluorescent protein (GFP)-positive cells from captured images using a Cytation5 automated fluorescence microscope (BioTek) and analyzed using the Gen5 data analysis software (BioTek). The half-maximal inhibitory concentration (IC_50_) of the MAbs or sera was calculated using a nonlinear regression analysis with GraphPad Prism software.

For the MAb combination experiment, the RBD (20 nM) MAb was combined with the NTD MAb or an Ebola virus glycoprotein-specific MAb (ADI-15878) as a negative control. While the RBD MAb was diluted 3-fold, the concentration of the NTD or the control MAb was kept constant at 3 nM. The MAb combinations were then tested for their neutralization capacity.

### SARS-CoV-2 neutralization assay.

The neutralization assay using authentic virus was performed as described previously (31). In brief, MAbs with an initial concentration of 100 nM were serially diluted, mixed with pretitrated amounts of SARS-CoV-2 (multiplicity of infection [MOI] = 0.2), and incubated for 1 h at 37°C and 5% CO_2_. The inoculum was added to Vero-E6 cell monolayers in 96-well plates and incubated for 1 h at 37°C and 5% CO_2_. For initial screening, a single concentration of 100 nM was used instead. The virus-serum inoculum was removed, cells were washed with PBS, and medium was added. At 24 h postinfection, cells were treated with 10% paraformaldehyde, washed with PBS, and permeabilized with 0.2% Triton-X for 10 min at room temperature. Cells were immunostained with SARS-CoV-1 nucleocapsid protein-specific antibody (Sino Biologic; catalog no. 40143-R001) and Alexa Fluor 488-labeled secondary antibody. Stained cells were imaged using an Operetta (Perkin-Elmer) high-content imaging instrument, and the number of infected cells was determined using Harmony software (Perkin-Elmer).

### Isolation of PBMCs.

Approximately 64 ml of whole blood collected from each donor using 8-ml BD Vacutainer CPT sodium heparin mononuclear cell preparation tubes was stored upright at room temperature for >1 h prior to centrifugation. Samples were centrifuged using a swinging bucket centrifuge at room temperature for 30 min at 4°C at 1,800 relative centrifugal force (RCF). The mononuclear cell layer was removed by using a pipette and pooled in a single tube for each donor. The total volume was brought to 45 ml with Mg^2+^- and Ca^2+^-free Hanks’ balanced salt solution (HBSS). Cells were resuspended by inverting the tubes and centrifuged at room temperature for 10 min at 330 RCF. After removing the supernatant, the cells were resuspended in 90% FBS supplemented with 10% dimethyl sulfoxide (DMSO) to a final concentration of 1 × 10^7^ cells per ml and stored at −150°C.

### Human ACE2 and SARS-CoV-2 spike antigens.

Prefusion-stabilized SARS-CoV-2 S-2P spike ectodomain (residues 1 to 1208) was expressed and purified as described previously (7). Plasmids encoding residues 1 to 305 of the SARS-CoV-2 spike with a C-terminal HRV3C cleavage site, monomeric Fc tag, and 8× His tag (SARS-CoV-2 NTD); residues 319 to 591 of the SARS-CoV-2 spike with a C-terminal HRV3C cleavage site, monomeric Fc tag, and 8× His tag (SARS-CoV-2 RBD-SD1); and residues 1 to 615 of hACE2 with a C-terminal 8× His tag and TwinStrepTag (hACE2) were transfected into FreeStyle-293F cells. Cell supernatants were harvested after 6 days, and expressed proteins were purified by affinity chromatography using a Superdex 200 Increase column (Cytiva). The SARS-CoV-2 NTD and RBD-SD1 proteins were purified using protein A resin (Pierce), whereas hACE2 protein was purified using StrepTactin resin (IBA). These proteins were then further purified by size exclusion chromatography using a buffer composed of 2 mM Tris (pH 8.0), 200 mM NaCl, and 0.02% NaN_3_. The SARS-CoV-2 S1 subunit (catalog no. S1N-C52H3) was purchased from Acro Biosystems.

### Sorting of SARS-CoV-2 spike-reactive single B cells.

B cells were purified and sorted as described previously (19). In short, B cells purified from donor PBMCs using the MACS human B-cell isolation kit (Miltenyi Biotec; catalog no. 130-091-151) were stained with a panel of antibodies: anti-human CD19 (phycoerythrin [PE]-Cy7; BioLegend catalog no. 302216), CD3 (peridinin chlorophyll protein [PerCP]-Cy5.5; BioLegend catalog no. 30040), CD8 (PerCP-Cy5.5; BioLegend catalog no. 344710), CD14 (PerCP-Cy5.5; Invitrogen catalog no. 45-0149-42), CD16 (PerCP-Cy5.5; BioLegend catalog no. 360712), IgM (BV711; BD Biosciences catalog no. 747877), IgD (BV421; BioLegend catalog no. 348226), IgA (AF-488; Abcam catalog no. Ab98553), IgG (BV605; BD Biosciences catalog no. 563246), CD27 (BV510; BD Biosciences catalog no. 740167), CD71 (allophycocyanin [APC]-Cy7; BioLegend catalog no. 334110), propidium iodide (PI), and an equimolar mixture of APC- and PE-labeled SARS-CoV-2 S-2P protein tetramers. BD FACS Aria II Fusion (BD Biosciences) was used for index sorting. The class-switched B cells were defined as CD19^+^ CD3^−^ CD8^−^ CD14^−^ CD16^−^ PI^−^ IgM^−^ IgD^−^ cells with a reactivity to APC- and PE-labeled SARS-CoV-2 S-2P tetramers. Single cells were sorted, and plates were stored at −80°C until further processing. Flow cytometry data were analyzed using FlowJo software.

### Amplification and cloning of IgG variable heavy and light chain genes.

Human antibody variable gene transcripts (VH, Vκ, Vλ) were amplified and cloned as previously described (34). Briefly, reverse transcription PCR (RT-PCR) (SuperScript IV enzyme [Thermo Scientific]) followed by nested PCR (HotStarTaq Plus DNA polymerase [Qiagen]) with cocktails of variable region and IgM-, IgD-, IgA-, and IgG-specific constant-region primers was performed. The next nested PCR was carried out to allow cloning by homologous recombination, and amplified gene transcripts were transformed into Saccharomyces cerevisiae (51). Finally, yeast cells were washed with sterile water, resuspended in selective media, and plated. The individual yeast clones were analyzed using Sanger sequencing.

### Expression and purification of human MAbs.

MAbs were expressed as full-length human IgG1 proteins in S. cerevisiae cultures, as previously described (34). Briefly, yeast cultures were grown in a 24-well format at 30°C and 80% relative humidity with shaking at 650 rpm in Infors Multitron shakers. Culture supernatants were harvested after 6 days of growth, and IgGs were purified by protein A affinity chromatography. IgGs bound to the agarose were eluted with 200 mM acetic acid with 50 mM NaCl (pH 3.5) and neutralized with 1/8 (vol/vol) 2 M HEPES (pH 8.0).

### BLI to assess MAb-antigen binding.

As previously described (34), for apparent equilibrium dissociation constant (*K_D_*App) determination the ForteBio Octet HTX instrument (Molecular Devices) (34) was used to measure the biolayer interferometry (BLI) kinetic of IgG binding to recombinant antigens. In short, the IgGs were captured on anti-human IgG (AHC) biosensors (Molecular Devices). For BLI measurements involving Strep-tagged antigens, the sensors were additionally incubated in a biocytin solution to saturate remaining Strep binding sites. After a 1-min baseline step, IgG-loaded biosensors were exposed for 180 s to the recombinant antigen. Next, the dissociation of the antigen from the biosensor surface was measured. For binding responses of >0.1 nm, data were aligned, interstep corrected to the association step, and fit to a 1:1 binding model using the ForteBio data analysis software, version 11.1.

### MAb competition assay using BLI.

Competitive binding of MAbs to the recombinant SARS-CoV-2 RBD with hACE2 was evaluated using the ForteBio Octet HTX instrument (Molecular Devices) as described previously (34). Briefly, IgGs were captured onto AHC biosensors (Molecular Devices) to achieve a sensor response between 1 and 1.4 nm followed by an inert IgG to occupy any remaining binding sites on the biosensor. The sensors were then equilibrated for a minimum of 30 min. The loaded sensors were additionally exposed to hACE2 for 90 s, prior to the binning analysis to assess any interactions between secondary molecules and proteins on the sensor surface. After a 60-s baseline step, association with the recombinant SARS-CoV-2 RBD was performed for 180 s and was finally followed by exposure to hACE2. The data were analyzed using the ForteBio data analysis software, version 11.0. The absence of binding by the secondary molecule indicates an occupied epitope (competitor), and binding indicates a noncompeting antibody.

### Epitope mapping using a yeast-display library.

Epitope mapping was done using a library of SARS-CoV-2 RBD point mutants displayed on the yeast surface as described previously (52). To select for mutants in the RBD-SD1 library with diminished binding to ADI-56443, the mutant RBD-SD1 library and wild-type (WT) RBD-SD1 yeast were incubated with ADI-56443 at its 80% effective concentration (EC_80_). Yeast cells from the library expressing hemagglutinin (HA) tag but with reduced ADI-56443 binding, compared to WT RBD SD1, were sorted by using a BD FACS Aria II (Becton Dickinson). The sorted cells were propagated, and the selection process was repeated to further enrich yeast cells encoding RBD mutants with reduced ADI-56443-binding. RBD sequences in the cell clones were sequenced, and those possessing single-amino-acid substitutions were cultured, protein-expression induced, and evaluated for their binding to ADI-56643 at EC_80_ by flow cytometry. Binding signal was normalized to that of the reference WT RBD-SD1 (set as 100%).

### Selection of neutralization-escape mutants of rVSV-SARS2.

Generation of rVSV-SARS2 that escaped neutralization with our top RBD (ADI-56443) and NTD (ADI-56479) MAbs was done as described previously (19). Briefly, rVSV-SARS2 was preincubated with 90% inhibitory concentrations (IC_90_s) of ADI-56443 (0.37 nM) and ADI-56479 (100 nM). Antibodies-virus mixtures were then applied to monolayers of Vero cells, and infection was allowed to proceed in the presence of the MAbs. Virus supernatants were harvested from the cells at 48 to 72 h postinfection (hpi) and passaged again by doubling the amount of antibody for subsequent passage. After 3 passages, MAb-resistant viruses were plaque purified, their phenotypes were confirmed by neutralization assay, and S gene sequences were determined by RT-PCR followed by Sanger sequencing as described previously (31).

### Assay for resistance to the generation of neutralization-escape rVSV-SARS2 mutants.

rVSV-SARS2 particles (MOI = 0.05) were incubated with IC_90_s of ADI-56443 (0.37 nM) and ADI-56479 (100 nM) for 1 h at room temperature, and the virus-antibody mixture was used to infect Vero cells in 6-well plates. Cells were imaged for enhanced GFP (eGFP) expression at 24 hpi. Virus supernatants were harvested at 48 hpi, and the amount of virus produced was determined by titration on Vero cells in the absence of MAbs as described previously (31).

### ELISA to detect binding of NTD MAbs to rVSV-SARS2 particles.

High-protein-binding 96-well ELISA plates (Corning) were coated with normalized amounts of purified parental or the mutant rVSV-SARS2 overnight at 4°C and blocked with 3% nonfat dry milk in phosphate-buffered saline (PBS-milk) for 1 h at 37°C. Plates were washed and incubated with biotinylated ADI-56479 at a concentration starting at 0.22 μg/ml with serial 3-fold dilutions in 1% PBS-milk supplemented with 0.1% Tween 20 for 1 h at 37°C. Plates were washed three times and incubated with streptavidin-horseradish peroxidase (HRP) (Pierce catalog no. 21130) diluted 1:3,000 in 1% PBS-milk supplemented with 0.1% Tween 20 for 1 h at 37C and detected using 1-Step Ultra TMB-ELISA substrate solution (Thermo Fisher Scientific). Plates were read using a Cytation 5 imager (BioTek) at 450 nm.
