# Supplementary figures and images for: A Combination of Receptor-Binding Domain and N-Terminal Domain Neutralizing Antibodies Limits the Generation of SARS-CoV-2 Spike Neutralization-Escape Mutants
Source: mBio. 2021 Oct 5;12(5):e02473-21. doi: 10.1128/mBio.02473-21 (PMC8546647; doi:10.1128/mBio.02473-21)

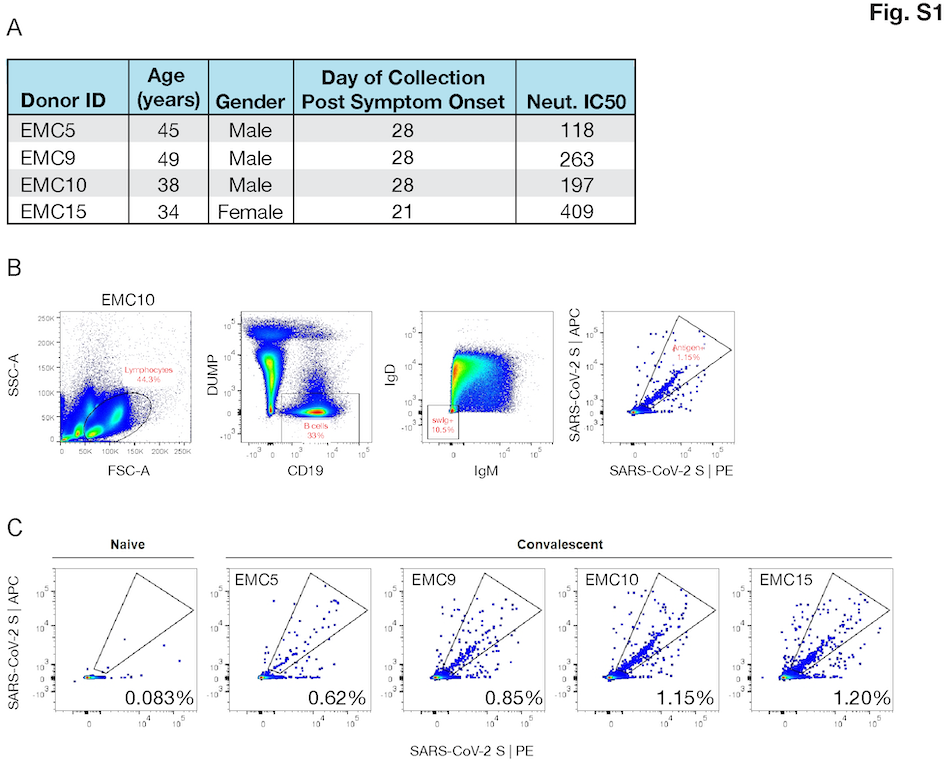

Supplement: FIG S1 [file mbio.02473-21-sf001.tif]

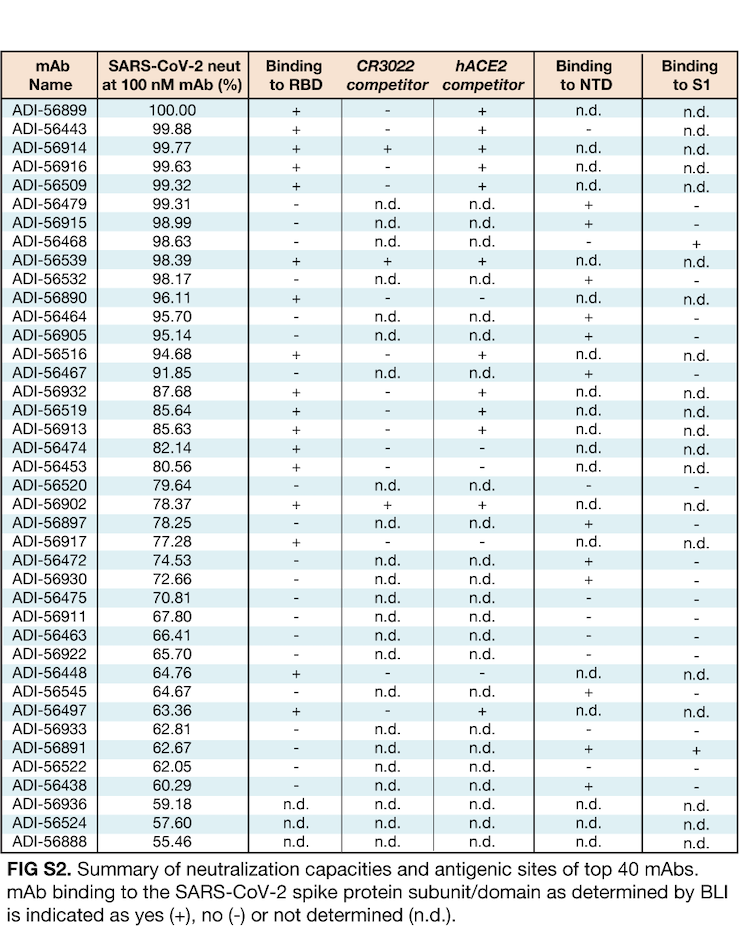

Supplement: FIG S2 [file mbio.02473-21-sf002.tif]
